# Supplementary material for: Outcomes of fenestrated endovascular abdominal aortic repair in distal entry tears of chronic debakey IIIb aortic dissection
Source: PLoS One. 2024 Feb 27;19(2):e0288218. doi: 10.1371/journal.pone.0288218 (PMC10898741; doi:10.1371/journal.pone.0288218)
Supplement: Attachment — Submitted filename: Response to Reviewers.docx [file pone.0288218.s003.docx]

Dear Editors and Reviewers,

Thank you for your decision and constructive comments on my manuscript. We have carefully considered the suggestion of Reviewer and make some changes. We have tried our best to improve and made some changes in the manuscript. The yellow part that has been revised according to your comments. Revision notes, point-to-point, are given as follows:

**1. The review’s comment:** I understand the indications you presented for the utilization of this technique, but could you elaborate more about the functional status of the abdominal organs before and after treatment (Lactate, Liver function, Kidney function)

**The authors’ answer:** According to the results of blood examination before and after operation, this operation (fenestrated endovascular abdominal aortic repair, F-EVAR) does not affect the function of abdominal organs. If patients are operated on because of abdominal organ ischemia, the abdominal organ function can be improved after operation.

**2. The review’s comment:** Can you elaborate if there is any place for this modality to be used in the acute setting in association with TEVAR?

**The authors’ answer:** F-EVAR can be used for emergency treatment as long as patient can wait 3-4 hours. Because we need to wait for 3-dimensional (3D) printed model of the aorta, which is usually 3-4 hours. Compared with commercial custom stents that need to wait 1-2 months, we think 3D printed aortic model-guided fenestrated stent is more suitable for emergency treatment.

**3. The review’s comment:** Was there any difference between the patient cohort who had aortic remodeling after this technique in terms of secondary prevention treatment such as taking b blockers?

**The authors’ answer:** In all the dissecting patients in our center, we routinely take b blockers orally, because there was a lot of evidence that long-term oral b blockers could improve the prognosis of dissecting patients. Therefore, it is not clear whether the absence of oral b blockers after F-EVAR will affect aortic remodeling.

**4. The review’s comment:** The manuscript needs an English revision.

**The authors’ answer:** We apologize for the poor language of our manuscript. We worked on the manuscript for a long time and the repeated addition and removal of sentences and sections obviously led to poor readability. We have now worked on both language and readability and have also involved native English speakers for language corrections. We really hope that the flow and language level have been substantially improved.

**5. The review’s comment:** The number of patients included in the study should be explain in the “Method section”, both in the abstract and in the text.

**The authors’ answer:** The number of patients included in the study has been described in the “Method section”, both in the abstract and in the text.

**6. The review’s comment:** In the section “Procedures”, the authors explain the technical procedure of construction of the prosthesis and the intraoperative procedure of implantation. In order to be clearer in the description of the different procedures in the text, I suggest to split the section in two subsections.

**The authors’ answer:** We have divided the section “Procedures” into two parts. The first part was the construction of fenestrated stents, and the second part was the implantation process in vivo.

**7. The review’s comment:** In the section “Outcome measures”, the sentence “Late outcomes were comprised of late aortic events, aortic remodelling, re-intervention, and late mortality” was repeated.

**The authors’ answer:** We are very grateful to the reviewers for their careful review and the repetitive sentences have been deleted.

**8. The review’s comment:** The “Table I” showed the type of procedures performed for each patient at the level of each abdominal vessel (celiac artery, superior mesenteric artery, left renal artery and right renal artery). The title of this table is “Dissection characteristics”. I suggest to choose a title that better reflects the contents of the table and to change the table style to be clearer to the reader. Moreover, the table should be more extensively explained in the text.

**The authors’ answer:** We have refined the title "Table I" and explained it in detail in the results section. The serial number of the Table is also adjusted.

**9. The review’s comment:** In the “Table II”, the variable “Reasons of F-EVAR” should be replaced by “Indication to F-EVAR”**.**

**The authors’ answer:** We have made corrections.

**10. The review’s comment:** The style of the Tables II and III should be changed, especially reporting more clearly what the values refer.

**The authors’ answer:** We have adjusted the order of the original Tables Ⅱ and Ⅲ. Separate the early outcome from the late outcome, so that the reader has a better understanding of what each variable represents.

**11. The review’s comment:** In the text the reference to “Figure 2”, which shows the CT images of a patient before and after the procedure, should not be referred in the section "Procedures", after the explanation of the interventional procedure. This reference should be moved to “Aortic remodelling” section.

**The authors’ answer:** We have put “Figure 2” into the to “Aortic remodelling” section.

**12. The review’s comment:** The “Figure 3” show the Kaplan-Meier curves of overall survival and aorta-specific survival. Detailed data on survival with patients at risk should be added.

**The authors’ answer:** We have added detailed data on survival with patients at risk.

**13. The review’s comment:** The bibliography includes some dated articles. I suggest to refer to more recent articles.

**The authors’ answer:** We have replaced those dated literatures with recent articles.

**14. The review’s comment:** In the section "Discussion", the authors should comment more extensively the comparison with other techniques available for the treatment of this pathology, including traditional surgery.

**The authors’ answer:** Other methods for the treatment of residual aortic dissection have been introduced in detail in the third paragraph of the discussion section. Traditional surgical procedures are not discussed in this article because this cohort was not suitable for patients undergoing traditional open surgery. It has been explained in the method section.

We tried our best to improve the manuscript and made some changes in the manuscript.

We appreciate for Editors/Reviewers’ warmwork earnestly, and hope that the correction will meet with approval. Once again thank you very much for your comments and suggestiongs.

Yours sincerely,

Wei Liu
