# Supplementary material for: Outcomes of fenestrated endovascular abdominal aortic repair in distal entry tears of chronic debakey IIIb aortic dissection
Source: PLoS One. 2024 Feb 27;19(2):e0288218. doi: 10.1371/journal.pone.0288218 (PMC10898741; doi:10.1371/journal.pone.0288218)
Supplement: Attachment — Submitted filename: Response to Reviewers.docx [file pone.0288218.s004.docx]

Dear Editors and Reviewers,

Thank you for your decision and constructive comments on my manuscript. We have carefully considered the suggestion of Reviewer and make some changes. We have tried our best to improve and made some changes in the manuscript. The yellow part that has been revised according to your comments. Revision notes, point-to-point, are given as follows:

**1. The review’s comment:** Minor proof-readig for some small errors in the text.

**The authors’ answer:** We are very grateful to the reviewers’ reminders and have corrected the errors in the manuscript.

**2. The review’s comment:** Please do indicate tipe (time?) uints in your KM curves.

**The authors’ answer:** We have made corrections.

**3. The review’s comment:** Table one incorporates table 2, please do corrct (correct?) the styling.

**The authors’ answer:** Thank you very much for reminding us that we have made adjustments.

**4. The review’s comment:** Table 2 (4?) endolieak (endoleak?) type?

**The authors’ answer:** We have added endoleak type in Table 4.

**5. The review’s comment:** In the “Procedures” section, would it be possible to provide more details on the creation of the 3D aortic template?

**The authors’ answer:** We have added our detailed steps to make the 3D aortic template in the “Procedures” section.

**6. The review’s comment:** In the “Baseline characteristics” section, is it possible to specify the patients’ symptoms?

**The authors’ answer:** We have made corrections.

**7. The review’s comment:** In Table 1 “Initial patient characteristics”, and particularly in the comorbidities section, is it possible to specify if there were patients with connective tissue disease?

**The authors’ answer:** These patients were not associated with connective tissue disease.

**8. The review’s comment:** Details regarding the procedures should be added in the “Operative details” section (for example the time needed to prepare the grafts and the duration of the entire procedures, how much contrast volume was used during the procedures and how many transfusions were performed in case of blood loss, and other information that may be useful…) Table 2 “Dissection characteristics with details of reconstruction of brunch vessels” should be completely modified as it is not very intelligible in this format

**The authors’ answer:** We have readjusted and simplified the " Table 2" and added a lot of surgery-related information.

**9. The review’s comment:** Have you ever had technical errors that led you to change your surgical strategy over the years?

**The authors’ answer:** We once encountered a case of left renal artery catheterization failure, which was rescued by parallel stent technique. It is described in detail in “ early outcomes ” section.

**10. The review’s comment:** Do you have any special precautions in case of severe aortic tortuosity?

**The authors’ answer:** We usually evaluate whether the distortion of the aorta can be operated on by endovascular surgery before operation. If it is not suitable for general open surgery, but so far, our center has not encountered this kind of situation.

We tried our best to improve the manuscript and made some changes in the manuscript.

We appreciate for Editors/Reviewers’ warmwork earnestly, and hope that the correction will meet with approval. Once again thank you very much for your comments and suggestiongs.

Yours sincerely,

Wei Liu
